# A Case of Recurrent Liver Injury-Associated Acute Pancreatitis (LIAAP)

**DOI:** 10.7759/cureus.65272

**Published:** 2024-07-24

**Authors:** Logan Oliver, Chuchu Liu, Brett Sadowski

**Affiliations:** 1 Internal Medicine, Naval Medical Center San Diego, San Diego, USA; 2 Gastroenterology, Naval Medical Center San Diego, San Diego, USA

**Keywords:** disseminated intravascular coagulation (dic), ischemic hepatopathy, drug-induced liver injury, acute pancreatitis, acute liver injury

## Abstract

Many etiologies of acute liver injury (ALI) include drug-induced liver injury (DILI), viral illness, and autoimmune disease. Acute pancreatitis is an uncommon though significant etiology of ALI caused by inflammation, fluid shifts, and ischemia secondary to microthrombi formation that can progress to liver failure if left untreated. We present a case of hypertriglyceridemia-induced pancreatitis resulting in liver injury-associated acute pancreatitis (LIAAP) and a concurrent consumptive coagulopathy consistent with an ischemic hepatopathy. Through treatment of her pancreatitis with intravenous insulin and plasmapheresis and subsequent transition to an oral regimen for her hypertriglyceridemia upon hospital discharge, the patient demonstrated full resolution of her ALI and coagulopathy. Through this case, we hope to highlight the importance of recognizing LIAAP and its underlying pathogenesis.

## Introduction

Acute pancreatitis is an uncommon etiology of acute liver injury (ALI), resulting from excessive activation of a systemic inflammatory response cascade that can result in multi-organ dysfunction [1]. We present a case of a 37-year-old female admitted for hypertriglyceridemia-induced pancreatitis and diabetic ketoacidosis (DKA) in the setting of medication nonadherence. Early into the patient’s admission, she developed a consumptive coagulopathy with markedly elevated transaminases consistent with an ischemic hepatopathy. With proper management of her pancreatitis and DKA, her coagulopathy resolved, and the transaminases normalized rapidly with clinical resolution.

## Case presentation

A 37-year-old female with insulin-dependent diabetes mellitus, hypertriglyceridemia, and recurrent pancreatitis presented to the emergency room with several days of mid-epigastric abdominal pain in the setting of medication nonadherence over the previous three weeks (to include her regimen of atorvastatin, fenofibrate, and icosapent ethyl for her hypertriglyceridemia). Following fluid resuscitation, laboratory evaluation confirmed hypertriglyceridemia-induced pancreatitis and DKA. Her initial laboratory evaluation on day 1 of her hospital admission is listed in Table 1 (of note, liver function testing was not obtained on the day of her hospital admission).

**Table 1:** Laboratory evaluation on day 1 of hospital admission cells/mm3 - cells per cubic millimeters; g/dL - grams per deciliter; cells/µL - cells per microliter; mmol/L - millimoles per liter; mg/dL - milligrams per deciliter; mmHg - millimeters of mercury PT - prothrombin time, PTT - partial thromboplastin time, INR - international normalized ratio

Parameter	Observed value	Reference range
White blood cells	4.0 cells/mm3	3,200-10,800 cells/mm3
Hemoglobin	14.5 g/dL	13.1-18.6 g/dL
Platelets	314 cells/µL	150,000-350,000 cells/µL
PT	13.2 seconds	12-15 seconds
PTT	26.9 seconds	25-35 seconds
INR	1.0	0.8-1.2
Fibrinogen	571 mg/dL	219-482 mg/dL
Serum sodium	130 mmol/L	136-145 mmol/L
Serum potassium	3.7 mmol/L	3.5-5.1 mmol/L
Serum chloride	108 mmol/L	98-107 mmol/L
Serum bicarbonate	6 mmol/L	21-32 mmol/L
Serum urea	7 mg/dL	7-18 mg/dL
Serum creatinine	0.4 mg/dL	0.7-1.3 mg/dL
Random blood glucose	214 mg/dL	70-100 mg/dL
Serum calcium	7.6 mg/dL	8.5-10.1 mg/dL
Serum phosphorus	<0.7 mg/dL	2.5-4.9 mg/dL
Serum magnesium	1.3 mg/dL	1.6-2.6 mg/dL
Serum triglycerides	3,952 mg/dL	50-199 mg/dL
Hemoglobin A1C	11.6%	4.8-5.6%
Venous blood gas lactic acid	1.48 mmol/L	0.9-1.7 mmol/L
Venous blood gas pH	7.24	7.31-7.41
Venous blood gas pCO_2_	22.0 mmHg	41-51 mmHg

She was subsequently treated with intravenous insulin and plasmapheresis, with rapid symptomatic improvement and resolution of her DKA. However, on day 3 of her admission, she demonstrated a marked ALI and coagulopathy without mental status changes. Her laboratory evaluation obtained on day 3 of her hospital admission is listed in Table 2.

**Table 2:** Laboratory evaluation on day 3 of hospital admission cells/mm3 - cells per cubic millimeters; g/dL - grams per deciliter; cells/µL - cells per microliter; mmol/L - millimoles per liter; mg/dL - milligrams per deciliter; IU/L - international units per liter; mmHg - millimeters of mercury PT - prothrombin time, PTT - partial thromboplastin time, INR - international normalized ratio

Parameter	Observed value	Reference range
White blood cells	3.4 cells/mm3	3200-10,800 cells/mm3
Hemoglobin	9.1 g/dL	13.1-18.6 g/dL
Platelets	32 cells/µL	150,000-350,000 cells/µL
PT	29.9 seconds	12-15 seconds
PTT	46.7 seconds	25-35 seconds
INR	2.9	0.8-1.2
Fibrinogen	166 mg/dL	219-482 mg/dL
Serum sodium	137 mmol/L	136-145 mmol/L
Serum potassium	4.1 mmol/L	3.5-5.1 mmol/L
Serum chloride	109 mmol/L	98-107 mmol/L
Serum bicarbonate	18 mmol/L	21-32 mmol/L
Serum urea	3 mg/dL	7-18 mg/dL
Serum creatinine	0.5 mg/dL	0.7-1.3 mg/dL
Random blood glucose	178 mg/dL	70-100 mg/dL
Serum calcium	7.4 mg/dL	8.5-10.1 mg/dL
Serum phosphorus	2.8 mg/dL	2.5-4.9 mg/dL
Serum magnesium	2.3 mg/dL	1.6-2.6 mg/dL
Serum total bilirubin	2.4 mg/dL	0.15-1.00 mg/dL
Serum direct bilirubin	1.6 mg/dL	0.2-0.3 mg/dL
Serum alkaline phosphatase	101 IU/L	40-129 IU/L
Serum alanine aminotransferase	5,308 IU/L	17-63 IU/L
Serum aspartate aminotransferase	>8404 IU/L	12-39 IU/L
Venous blood gas pH	7.36	7.31-7.41
Venous blood gas pCO_2_	34.1 mmHg	41-51 mmHg

A thorough workup of her ALI, including abdominal computed tomography (CT), ultrasound imaging, and an autoimmune and infectious laboratory evaluation, were ultimately unremarkable. Outside hospital records from her last hospitalization for pancreatitis approximately six months prior were obtained, and it was noted that a liver biopsy was taken at the time with evidence of lobular hepatocyte injury without fibrosis. Because of this, a decision was made to forego a subsequent liver biopsy, and no direct interventions for the ALI were performed.

After this exhaustive workup, drug-induced liver injury (DILI) and ischemic hepatopathy remained the most likely etiologies. With these possibilities came a clinical dilemma related to the management of her recurrent pancreatitis: how should the lipid-lowering therapy which could at least in rare cases cause liver damage be handled? During her previous episodes, she was presumed to have DILI secondary to her hypertriglyceridemia medication regimen of atorvastatin, fenofibrate, and icosapent ethyl, and discontinued this regimen for a short duration. However, she had intermittent periods of medication compliance over the next several months, with full discontinuation of all her home medications three weeks prior due to gastrointestinal distress.

With continued treatment of her DKA and pancreatitis while inpatient, her transaminases downtrended considerably with near resolution of her coagulopathy by the date of her hospital discharge (day 8). Her laboratory evaluation obtained on day 8 of her hospital admission is listed in Table 3.

**Table 3:** Laboratory evaluation on day 8 of hospital admission and date of discharge cells/mm3 - cells per cubic millimeters; g/dL - grams per deciliter; cells/µL - cells per microliter; mmol/L - millimoles per liter; mg/dL - milligrams per deciliter; IU/L - international units per liter PT - prothrombin time, PTT - partial thromboplastin time, INR - international normalized ratio

Parameter	Observed value	Reference range
White blood cells	5.8 cells/mm3	3,200-10,800 cells/mm3
Hemoglobin	9.9 g/dL	13.1-18.6 g/dL
Platelets	147 cells/µL	150,000-350,000 cells/µL
PT	13.8 seconds	12-15 seconds
PTT	32.1 seconds	25-35 seconds
INR	1.0	0.8-1.2
Fibrinogen	501 mg/dL	219-482 mg/dL
Serum sodium	138 mmol/L	136-145 mmol/L
Serum potassium	3.5 mmol/L	3.5-5.1 mmol/L
Serum chloride	101 mmol/L	98-107 mmol/L
Serum bicarbonate	28 mmol/L	21-32 mmol/L
Serum urea	5 mg/dL	7-18 mg/dL
Serum creatinine	0.4 mg/dL	0.7-1.3 mg/dL
Random blood glucose	180 mg/dL	70-100 mg/dL
Serum calcium	9.5 mg/dL	8.5-10.1 mg/dL
Serum phosphorus	4.0 mg/dL	2.5-4.9 mg/dL
Serum magnesium	2.0 mg/dL	1.6-2.6 mg/dL
Serum total bilirubin	1.18 mg/dL	0.15-1.00 mg/dL
Serum alkaline phosphatase	172 IU/L	40-129 IU/L
Serum alanine aminotransferase	1,630 IU/L	17-63 IU/L
Serum aspartate aminotransferase	70 IU/L	12-39 IU/L
Serum triglycerides	264 mg/dL	50-199 mg/dL

She was subsequently discharged to home with her previous home regimen of atorvastatin, fenofibrate, and icosapent ethyl for her hypertriglyceridemia, along with her previous home insulin regimen and close follow-up with an endocrinologist. At her gastroenterology outpatient follow-up appointment two weeks later, repeat complete blood count and liver function testing demonstrated near total resolution of her ALI and coagulopathy, with her laboratory evaluation listed in Table 4.

**Table 4:** Laboratory evaluation two weeks after hospital discharge cells/mm3 - cells per cubic millimeters; g/dL - grams per deciliter; cells/µL - cells per microliter; mmol/L - millimoles per liter; mg/dL - milligrams per deciliter; IU/L - international units per liter PT - prothrombin time, INR - international normalized ratio

Parameter	Observed value	Reference range
White blood cells	6.3 cells/mm3	3,200-10,800 cells/mm3
Hemoglobin	11.3 g/dL	13.1-18.6 g/dL
Platelets	411 cells/µL	150,000-350,000 cells/µL
PT	13.4 seconds	12-15 seconds
INR	1.0	0.8-1.2
Serum sodium	140 mmol/L	136-145 mmol/L
Serum potassium	3.7 mmol/L	3.5-5.1 mmol/L
Serum chloride	103 mmol/L	98-107 mmol/L
Serum bicarbonate	27 mmol/L	21-32 mmol/L
Serum urea	16 mg/dL	7-18 mg/dL
Serum creatinine	0.5 mg/dL	0.7-1.3 mg/dL
Random blood glucose	207 mg/dL	70-100 mg/dL
Serum calcium	10.3 mg/dL	8.5-10.1 mg/dL
Serum phosphorus	3.3 mg/dL	2.5-4.9 mg/dL
Serum magnesium	1.6 mg/dL	1.6-2.6 mg/dL
Serum total bilirubin	0.63 mg/dL	0.15-1.00 mg/dL
Serum direct bilirubin	0.3 mg/dL	0.2-0.3 mg/dL
Serum alkaline phosphatase	78 IU/L	40-129 IU/L
Serum alanine aminotransferase	97 IU/L	17-63 IU/L
Serum aspartate aminotransferase	36 IU/L	12-39 IU/L
Serum triglycerides	158 mg/dL	50-199 mg/dL

Given the clinical and near total laboratory resolution with continued medication compliance, her initial presentation ultimately suggested an ALI secondary to a transient ischemic hepatopathy caused by her acute pancreatitis.

## Discussion

Acute pancreatitis is a common medical condition often requiring hospitalization with simple fluid resuscitation, pain control, and bowel rest. However, delayed or inadequate treatment can result in severe acute pancreatitis, which can result in multiple organ failures, including liver injury-associated acute pancreatitis (LIAAP) and subsequent liver failure [2].

Though the exact mechanism of LIAAP is not clearly defined, it is theorized that excessive activation of a systemic inflammatory response cascade leads to liver ischemia due to an acute decrease in liver perfusion [1,3]. In the pathophysiology of acute pancreatitis, activated digestive enzymes induce neutrophils to release inflammatory factors which can result in multiple organ dysfunction [4]. It is believed that increased activation of tumor necrosis factor-α results in the activation of neutrophils and subsequent release of IL-1, IL-6, IL-8, and other cytokines contributing to the inflammatory state leading to hepatocellular injury [5]. Given its proximity to the pancreas, the liver is often the first extrapancreatic organ attacked by these inflammatory mediators.

In addition, acute pancreatitis can result in low blood volume caused by this proinflammatory state [6]. In acute pancreatitis, there is typically a 50% decrease in portal venous blood flow [7]. This marked drop in volume results in subsequent poor liver perfusion, clinically manifesting in ALI and possible progression to acute liver failure [8].

The treatment of LIAAP is the first and foremost treatment of the underlying acute pancreatitis. Typical treatment includes fluid resuscitation, which helps to further increase portal venous blood flow that was previously reduced due to inflammatory mediators [9]. In addition, it is essential to provide adequate pain control and bowel rest, along with a slow addition of nutritional supplementation [10].

Several clinical features can assist in distinguishing LIAAP from DILI. As in our patient, the swift downtrend of her transaminases with the treatment of her pancreatitis, along with her laboratory values notable for hypophosphatemia, hyperglycemia, and a coagulopathy consistent with a mild disseminated intravascular coagulation presentation, all suggest an ischemic etiology [11].

## Conclusions

Though acute pancreatitis is a common presentation in the inpatient hospital setting, it is important to recognize this disease process as a possible cause of ALI and acute liver failure. Additionally, one should consider an ischemic hepatopathy etiology in patients admitted for acute pancreatitis who demonstrate an ALI with consumptive coagulopathy. With this case, we hope to highlight the importance of monitoring for liver organ dysfunction in acute pancreatitis and its underlying pathogenesis.
